# Total Thyroidectomy vs. Lobectomy in Papillary Thyroid Microcarcinoma: A Contested Gold Standard

**DOI:** 10.3390/jpm15070324

**Published:** 2025-07-18

**Authors:** Enrico Battistella, Luca Pomba, Riccardo Toniato, Andrea Piotto, Antonio Toniato

**Affiliations:** 1 Endocrine Surgery Unit, Department of Surgery, Veneto Institute of Oncology, IOV-IRCCS, Via Gattamelata 64, 35128 Padua, Italy; luca.pomba@iov.veneto.it (L.P.); andrea.piotto@iov.veneto.it (A.P.); antonio.toniato@iov.veneto.it (A.T.); 2 School of Medicine, University of Padua, Via Giustiniani 2, 35128 Padua, Italy; riccardo.toniato@studenti.unipd.it

**Keywords:** papillary thyroid microcarcinoma, papillary thyroid tumor, total thyroidectomy, hemithyroidectomy, active surveillance

## Abstract

**Background**: Papillary thyroid microcarcinoma (PTMC), a subtype of papillary thyroid carcinoma ≤ 1 cm in diameter, has shown a marked increase in incidence in recent decades, largely due to the widespread use of neck ultrasonography and fine needle aspiration cytology. Despite its generally indolent course, optimal management of PTMC remains controversial, with treatment strategies ranging from active surveillance to total thyroidectomy. **Methods**: This retrospective study analyzes five years of experience at a single tertiary care center, including 130 patients diagnosed with PTMC following thyroid surgery between July 2018 and December 2023. Clinical, cytological, and pathological data were collected and analyzed to identify factors influencing surgical decision-making and postoperative outcomes. Patients underwent either total thyroidectomy or hemithyroidectomy, with central and lateral lymph node dissection performed as indicated. Follow-up included clinical and biochemical surveillance for a mean duration of 3 years. **Results**: Total thyroidectomy was performed in 89.3% of patients, while hemithyroidectomy was limited to 10.7%. Multifocality was observed in 26.1% of cases, with bilateral involvement in 17.7%. Occult lymph node metastases were found in 14.6% (central compartment) and 3.8% (lateral neck). Postoperative radioactive iodine therapy was administered in 23.8% of patients. At final follow-up, 90.7% were disease-free. No significant predictors of recurrence or adverse outcomes were identified, though multifocality and lymph node involvement influenced surgical planning. **Conclusions**: Our findings support a risk-adapted surgical approach to PTMC, favoring total thyroidectomy in patients with suspicious or multifocal disease to avoid reoperation. While active surveillance and minimally invasive techniques are emerging, total thyroidectomy remains a safe and effective strategy in selected cases. Prospective, multicenter studies are needed to further refine management guidelines for this increasingly common thyroid malignancy.

## 1. Introduction

Papillary carcinoma is the most common histologic type of malignancy originating from the thyroid gland [1,2]. The proportion of papillary thyroid microcarcinomas (PTMCs) increased from 25% of new thyroid cancer diagnoses in 1999 to 39% in 2009 [3,4]. The incidence of thyroid cancer has risen sharply over the past three decades, primarily due to the widespread use of ultrasonography (US) and US-guided fine needle aspiration cytology (FNAC) [4].

PTMC refers to a subtype of papillary thyroid carcinoma (PTC), characterized by a tumor size of 1 cm or less [5]. Despite its generally favorable prognosis, the increasing incidence of PTMC has raised important questions regarding its clinical management, biological behavior, and appropriate treatment strategies. These tumors are, in fact, the most common type of thyroid cancer found in up to 36% of cases in autopsy studies [5,6].

PTMC can be classified as “incidental” when it is discovered in the histological analysis of a thyroid gland removed for benign conditions (e.g., multinodular goiter or Basedow’s disease). Alternatively, it can be described as “occult” when it is undetectable during preoperative clinical examination and only indirectly diagnosed due to the presence of metastatic lymph nodes [7].

While many patients with PTMC exhibit indolent disease with minimal risk of metastasis, others may experience recurrence or complications. This paradox has led to ongoing debate within the medical community regarding the optimal approach to diagnosis, surveillance, and treatment.

The American Thyroid Association (ATA) and the Italian Association of Medical Oncology (AIOM) guidelines address the management of low-risk thyroid carcinoma [8,9]. A primary aim of these guidelines is to minimize the potential harm of overtreatment in the majority of patients who are at low risk of disease-specific mortality and morbidity, while ensuring appropriate treatment and follow-up for higher-risk individuals. Indeed, a less aggressive surgical approach, such as hemithyroidectomy, should be considered for patients with low-risk PTMC [8,9,10].

This article presents a comprehensive review of our center’s five-year experience in managing PTMC, with a particular focus on the surgical approach adopted. It aims to provide an in-depth analysis of the various risk factors that must be carefully evaluated both during the preoperative decision-making process and throughout the intraoperative period. By reflecting on the outcomes and challenges encountered over this period, we seek to offer valuable insights into optimizing surgical planning, improving patient selection, and enhancing intraoperative strategies to reduce complications and improve overall results.

## 2. Materials and Methods

### 2.1. Patient Population and Study Design

This retrospective observational study included patients diagnosed with papillary thyroid microcarcinoma (PTMC) who underwent hemithyroidectomy or total thyroidectomy at our institution between July 2018 and December 2023. Inclusion criteria comprised histopathological confirmation of PTMC (maximum tumor diameter ≤ 1 cm) in the surgical specimen.

For each case, clinical and pathological data were collected and analyzed. The variables considered included:

- Patient demographics—age and sex.
- Thyroid background—presence of chronic lymphocytic thyroiditis or nodular goiter.
- Risk factors—prior exposure to ionizing radiation and family history of thyroid disorders or malignancies.
- Comorbidities—relevant associated medical conditions.
- Surgical details—indication for surgery and type of procedure performed (hemithyroidectomy vs. total thyroidectomy).

Surgical planning was guided by several factors, including the cytological classification of the nodule, the presence of concurrent thyroid conditions (such as Hashimoto’s thyroiditis, toxic or multinodular goiter, or Graves’ disease), patient age, and a family history of thyroid carcinoma. In most cases of solitary nodules smaller than 1 cm with cytology classified as TIR4 or TIR5, a hemithyroidectomy was the preferred approach. Conversely, if one or more of the mentioned risk factors were identified, total thyroidectomy was considered the more appropriate surgical option. In patients presenting with multinodular goiter and either a confirmed malignancy or the presence of a high-risk nodule, total thyroidectomy remains the standard surgical approach.

In cases where metastatic involvement of cervical lymph nodes was identified preoperatively through imaging or clinical assessment, a lateral cervical lymph node dissection was carried out as part of the surgical management.

The exclusion criteria for this study included the following: patients under 18 years of age, papillary thyroid carcinomas larger than 1 cm in diameter, and histological diagnoses other than papillary carcinoma (i.e., other tumor histotypes).

Postoperative follow-up was conducted at 3, 6, and 12 months and, subsequently, on an annual basis. The mean follow-up duration was 3 years (range: 14 months to 7 years). Surveillance included clinical examination, serum thyroid function tests (TSH, free T3, free T4), serum thyroglobulin (Tg), anti-thyroglobulin antibodies (TgAb), and high-resolution cervical ultrasonography.

Pathological staging was performed by our two dedicated, experienced pathologists in accordance with the AJCC 8th edition TNM classification system. In patients who underwent initial lobectomy with subsequent completion thyroidectomy, adjuvant radioactive iodine (RAI) therapy with I-131 and/or reoperation were documented when applicable.

### 2.2. Statistical Analysis

Descriptive statistics were used to summarize the data. Categorical variables were expressed as absolute numbers and percentages, while continuous variables were reported as means ± standard deviation (SD), medians, and interquartile ranges (IQRs) where appropriate.

Comparisons between groups were performed using the Chi-square test or Fisher’s exact test for categorical variables. For continuous variables, Student’s *t*-test or non-parametric tests (Wilcoxon rank-sum or Kruskal–Wallis test) were applied based on distribution normality. A *p*-value < 0.05 was considered statistically significant. All statistical analyses were carried out using SAS software, version 9.4 (SAS Institute Inc., Cary, NC, USA).

### 2.3. Ethical Aspects

This study was conducted in compliance with the principles of the Declaration of Helsinki and adhered to Good Clinical Practice (GCP) guidelines. Institutional approval was obtained from the Ethics Committee of the Veneto Oncology Institute (IOV-IRCCS) on 26 July 2023 (protocol number: IOV-ENDCH-01-2023). Given the retrospective nature of the study, informed consent was waived in accordance with applicable local regulations.

## 3. Results

Between July 2018 and December 2023, a total of 1930 patients underwent thyroid surgery at our institution. Among them, 130 patients (6.7%) had histopathological confirmation of papillary thyroid microcarcinoma (PTMC) and were included in this study. The mean age of the cohort was 53.1 years (range: 19–85 years). Of the 130 patients, 98 were female (75.4%) and 32 were male (24.6%), yielding a female-to-male ratio of approximately 3:1.

Thyroid dysfunction was present in 84 patients (64.6%). Histopathological evidence of chronic lymphocytic thyroiditis was found in 30 patients (23.1%), and multinodular goiter was reported in 54 patients (41.5%). A family history of thyroid cancer was noted in 17 patients (13.1%).

The surgical indications were as follows: multinodular goiter in 35 patients (26.9%), Graves’ disease in 11 patients (8.5%), single indeterminate thyroid nodules (TIR3) in 30 patients (23.1%), highly suspicious nodules (TIR4) in 29 patients (22.3%), and cytologically confirmed thyroid carcinoma (TIR5) in 25 patients (19.2%). Patients with benign thyroid disease underwent total thyroidectomy primarily for compressive symptoms, dysphagia, or due to the presence of indeterminate nodules in the setting of thyroiditis.

Total thyroidectomy was performed in 116 patients (89.3%), while hemithyroidectomy was carried out in 14 patients (10.7%). Completion thyroidectomy was required in two cases following initial lobectomy.

Central compartment (Level VI) lymph node dissection was performed in 95 patients (73.0%), while lateral neck (Levels II–V) dissection was conducted in 5 patients (3.8%). Occult lymph node metastases in the central compartment were identified in 19 patients (14.6%) and in the lateral compartment in 5 patients (3.8%). Evidence of angioinvasion or perineural invasion was observed in 17 cases (13.1%).

Histopathological analysis revealed that all patients had T1a tumors (≤1 cm) according to the 8th edition of the American Joint Committee on Cancer (AJCC) TNM staging system. Multifocal PTMC was identified in 34 patients (26.1%), with bilateral involvement observed in 23 cases (17.7%). The BRAFV600E mutation was detected in five patients (3.8%).

Postoperative radioactive iodine (I-131) therapy was administered to 31 patients (23.8%) based on risk stratification.

At the last follow-up, 118 patients (90.7%) were disease-free with undetectable serum thyroglobulin levels, 10 patients (7.7%) had elevated serum thyroglobulin without evidence of recurrence, and 2 patients (1.6%) had died from unrelated causes.

The results obtained are summarized in Table 1.

## 4. Discussion

Papillary thyroid microcarcinomas (PTMCs), defined as papillary thyroid carcinomas ≤ 1 cm in diameter, have shown a marked increase in incidence in recent decades, with consistently excellent reported prognosis [8,9]. This rise is largely attributed to improved imaging modalities, particularly high-resolution neck ultrasonography, which has enhanced the detection of subclinical and asymptomatic thyroid nodules [3,4].

Despite their typically indolent course, the optimal management of PTMC remains a subject of ongoing investigation and clinical debate. Historically, surgical treatment, such as lobectomy or total thyroidectomy, was considered the standard approach. However, alternative strategies such as active surveillance have gained acceptance, particularly following the pioneering work by Ito et al. [11]. A recent survey by Sugitani et al. reported that over 50% of patients with low-risk PTMC in Japan are managed with active surveillance, which has been shown to positively impact quality of life and reduce healthcare costs [12].

Nevertheless, implementation of active surveillance varies globally. In our view, such an approach requires more rigorous diagnostic protocols than those typically adopted in Europe. In particular, the more conservative indications for fine-needle aspiration cytology (FNAC) may limit the early identification of a large number of PTMC cases [13].

In our retrospective cohort, we included all patients who underwent thyroid surgery between July 2018 and December 2023 and were found to have PTMC on final histology. Although no significant predictors of adverse outcomes were identified on univariate or multivariate analysis, likely due to the low number of such events, several findings warrant discussion.

Multifocal PTMC was detected in 34 patients (26.1%), with bilateral involvement observed in 23 cases (17.7%), despite surgery being initially indicated for a presumed solitary lesion. Multifocality in papillary thyroid carcinoma (PTC) is well-documented, and the presence of a single lesion does not exclude contralateral disease. The prognostic implications of multifocal PTMC remain controversial [14,15]. In a retrospective study of 109 patients with T1–T3N0Mx PTC, Mazur et al. found no reliable predictors of multifocality; however, ultrasonographic features may offer some predictive value [16]. If hemithyroidectomy had been performed as the initial intervention, approximately 20 patients (15.4%) in our series would have likely required completion thyroidectomy due to synchronous lesions in the contralateral lobe.

From our analysis, we identified 12 cases (9.2%) in which the treatment administered could be classified as overtreatment. In these instances, the extent of surgical intervention exceeded what was clinically necessary, as a thyroid lobectomy alone would have been sufficient to manage the condition effectively. This suggests a potential area for improving treatment protocols to avoid unnecessary total thyroidectomies, thereby reducing the risk of associated complications and improving overall patient outcomes.

Another contentious topic involves the significance of central neck lymph node metastases in PTMC. According to the American Thyroid Association (ATA) guidelines, PTMC with ≤5 micrometastases (≤2 mm in size) are still classified as low-risk, with an estimated recurrence risk of around 5% [8]. Occult central compartment metastases are reported in up to 31% of patients with PTC and are typically clinically insignificant [8,9,17]. In our study, 19 patients (14.6%) had central compartment metastases, and 5 patients (3.8%) had lateral compartment involvement. These findings influenced the decision to proceed with postoperative radioiodine ablation. For this reason, we advocate for total thyroidectomy when suspicious lymphadenopathy is encountered intraoperatively.

Mansour et al. highlighted the prognostic value of the lymph node ratio (LNR), defined as the number of metastatic lymph nodes divided by the total number examined, demonstrating that an LNR > 0.3 is an independent predictor of locoregional recurrence in N1-stage PTC [18]. Similarly, Ahn et al. supported prophylactic central neck dissection during thyroidectomy in PTMC, as it reduced recurrence rates while maintaining procedural safety [19].

Our surgical decision-making was guided by a risk-adapted approach in accordance with the latest NCCN guidelines [4]. Surgical planning considered cytological classification, the presence of coexisting thyroid pathology (e.g., Hashimoto thyroiditis, toxic or multinodular goiter, Graves’ disease), patient age, and family history of thyroid cancer. In cases of solitary nodules < 1 cm with cytology consistent with TIR4 or TIR5, hemithyroidectomy was generally favored. However, when any of the aforementioned risk factors were present, total thyroidectomy was preferred. Notably, many patients opted for upfront total thyroidectomy to avoid the potential need for reoperation in the event of adverse histological findings [20,21].

In our series, 130 patients were included, and most underwent total thyroidectomy for one or more of the reasons outlined above. The literature reflects an ongoing debate on this issue [22,23]. A multicenter study demonstrated favorable outcomes in patients with low-risk PTMC treated with lobectomy and no adjuvant radioiodine therapy over a five-year follow-up period [24]. Stimulated thyroglobulin levels remained low, and no distant metastases occurred. In contrast, a study of 2784 patients with differentiated thyroid carcinoma (DTC), including 86% with PTC, found that total thyroidectomy was associated with improved survival in high-risk patients [22]. Furthermore, data from the National Cancer Database involving 52,173 PTC cases revealed that tumors ≥ 1 cm treated with total thyroidectomy had significantly lower recurrence rates and improved survival compared to lobectomy [25].

In our cohort, 31 patients (23.8%) underwent postoperative radioiodine (I-131) therapy. For these individuals, an initial hemithyroidectomy would have likely necessitated a subsequent completion thyroidectomy, a procedure often complicated by fibrosis, adhesions, and altered anatomy resulting from the primary surgery. These challenges can increase operative time, risk of complications, and patient morbidity. Consequently, this observation supports our rationale for favoring total thyroidectomy as the initial surgical approach in appropriately selected patients, aiming to minimize the need for reoperation and optimize oncological outcomes.

Emerging techniques such as thermal ablation under ultrasound guidance offer a minimally invasive alternative for selected patients with PTMC. These procedures involve percutaneous delivery of thermal energy via fine needles to destroy tumor tissue while sparing adjacent thyroid parenchyma. Early studies report promising outcomes in terms of tumor control and preservation of thyroid function, though long-term data are still needed [26].

This study has several limitations. First, the number of patients treated with hemithyroidectomy was relatively small compared to those undergoing total thyroidectomy, limiting subgroup analysis. Second, the retrospective nature of the study introduces potential selection bias. Future prospective, multicenter studies with longer follow-up and randomized designs although challenging due to ethical and logistical constraints are essential to clarify the optimal management of PTMC.

## 5. Conclusions

The management landscape of papillary thyroid microcarcinoma (PTMC) is rapidly evolving. Current surgical guidelines appear increasingly uncertain, reflecting a broader shift toward less invasive and more personalized treatment strategies, though a universally accepted standard has yet to be established. Therapeutic options now include active surveillance, thermal ablation, and surgical intervention each with its own indications and limitations. Ongoing prospective studies and long-term outcome data will be crucial in refining the optimal approach to this increasingly prevalent diagnosis.

Nevertheless, surgical planning must take into account all clinical, cytological, and radiological variables that may suggest bilateral disease involvement. A thorough intraoperative assessment is essential, particularly when lymph node involvement or multifocality is suspected. Importantly, if surgery is pursued, the avoidance of completion thyroidectomy should be a strategic goal. Reoperation increases surgical risk due to scar tissue, altered anatomy, and a higher incidence of complications such as recurrent laryngeal nerve injury or hypoparathyroidism. Therefore, the initial surgical approach must be carefully calibrated, weighing clinical factors, patient preferences, and institutional expertise.

## Figures and Tables

**Table 1:** The results obtained are summarized in this table.

Patients’ Population Characteristics	Total of Patients = 130 Patients	Ongoing Disease = 10 Patients	Statistical Analyses *p*-Value
Gender	Female = 98 (75.4%)	Female = 6 (60%)	N.S.
	Male = 32 (24.6%)	Male = 4 (40%)	
Mean Age	53.1 years (Minimum 19, Maximum 85 years)	50 years	N.S.
Indication of surgery: • Multinodular Goiter • Basedow Disease • Cytologically indeterminate nodule • Thyroid carcinoma			N.S.
	35 (26.9%)		
	
	11 (8.5%)		
	
	30 (23.1%)	2 (20%)	
	
	
	TIR4 (29 (22.3%))	4 (40%)	
	TIR5 (25 (19.2%))	4 (40%)	
Type of surgery: • Total thyroidectomy • Lobectomy			N.S.
	116 (89.3%)	8 (80%)	
	
	14 (10.7%)	2 (20%)	
Lymph node dissection	C.C. (95 (73%))	8 (80%)	N.S.
	L.C. (5 (3.8%))	2 (2%)	
Unifocal tumor	96 (74.1%)	5 (%)	N.S.
Multifocal tumor	34 (26.1%) → bilateral multifocal tumor = 23 (17.7%)	5 (50%)	N.S.
Lymph nodes metastases	C.C. → 19 (14.6%) L.C. → 5 (3.8%)	5 (50%) 3 (30%)	N.S.
Neurovascular invasion	17 (13.1%)	4 (40%)	N.S.
131-Iodine treatment	31 (23.8%)	10 (100%)	N.S.

L.C. = Latero-cervical compartment, C.C. = central compartment, N.S. = non-significant.

## Data Availability

Data could be found in the patients’ medical records.
